# Investigator Argus X-12 study on the population of northern Croatia

**DOI:** 10.1590/1678-4685-GMB-2015-0261

**Published:** 2016-10-20

**Authors:** Josip Crnjac, Petar Ozretić, Siniša Merkaš, Martina Ratko, Mateja Lozančić, Marina Korolija, Maja Popović, Gordan Mršić

**Affiliations:** 1 University Department for Forensic Sciences, University of Split, Split, Croatia; 2 Ruđer Bošković Institute, Zagreb, Croatia; 3 Forensic Science Centre "Ivan Vučetić", Zagreb, Croatia; 4 Faculty of Veterinary Medicine, University of Zagreb, Zagreb, Croatia

**Keywords:** X-STR, forensic genetics, Investigator® Argus X-12, population genetics, northern Croatia

## Abstract

X chromosome STR typing has emerged recently as a powerful tool, complementary to autosomal STR typing, in solving complex forensic and missing person cases. Investigator® Argus X-12 is a commercial product that allows co-amplification of 12 X chromosomal markers belonging to four linkage groups (LGs). In this study, we analyzed by capillary electrophoresis blood samples from 100 females and 102 males from a population of northern Croatia. Statistical analysis included calculation of allele and haplotype frequencies, as well as forensic parameters. The most informative marker for the northern Croatia population was DXS10135 with PIC=0.9211 and a total of 27 alleles. The least polymorphic marker was DXS8378 with 6 alleles. The proportion of observed haplotypes from the number of possible haplotypes varied from 2.74–8.57% across all LGs, with LG1 being the most informative. Of the 11 tested world populations compared to the population of northern Croatia, significant differences in genetic distance (F_ST_) were found for Greenlandic and all non-European populations. We found that all tested markers are in HWE and can thus be used for match probability calculation. Because of high combined power of discrimination in both men and women, Investigator® Argus X-12 is applicable for the northern Croatia population in routine forensic casework.

## Introduction

Autosomal short tandem repeat (STR) testing has been by far the most important tool in DNA forensics for the last 25 years. Its individualization power today exceeds values of one in a trillion. As an additional STR typing tool, Y chromosome STRs have been introduced for deficiency paternity and rape cases, as well as missing person identification (Diegoli, 2014). Similarly, X chromosome STR (X-STR) typing is also proving to be a powerful complementary method that can aid forensic experts in solving complex forensic cases including deficiency paternity testing, identification of human remains and in any pedigree testing involving at least one female (Szibor *et al*., 2003; Branicki *et al*., 2008; Gomes *et al*., 2012; Diegoli, 2014). Since females have two X chromosomes, a certain level of homologous recombination can occur during meiosis. Alleles at linked loci can therefore be separated and inherited in F_1_ generation either as haplotypes in males or as a part of one homologue in females. Linkage disequilibrium (LD), which measures co-segregation of closely located alleles in a pedigree at the population level, should therefore be inferred from haplotype frequencies. LD is of great importance for casework, because it has an effect on probabilities calculation in paternity cases, and subsequent interpretation of the results (Tillmar *et al*., 2008).

A new multiplex kit has been placed on the market under the name Investigator® Argus X-12 Kit (Qiagen GmbH, Hilden, Germany) which is an upgrade of MenType® Argus X-8 Kit. This kit has 12 X-STR markers arranged in four linkage groups (LG), each comprising three markers: linkage group 1 (LG1) (DXS10148, DXS10135, DXS8378), linkage group 2 (LG2) (DXS7132, DXS10079, DXS10074), linkage group 3 (LG3) (DXS10103, HPRTB, DXS10101), and linkage group 4 (LG4) (DXS10146, DXS10134, DXS7423). Linkage and linkage disequilibrium was calculated for all loci within each of the four linkage groups and validated for forensic use in multiple studies (Hering *et al*., 2006a, b; Edelmann *et al*., 2008; Hundertmark *et al*., 2008; da Silva *et al*., 2010; Rodig *et al*., 2010). These loci segregate together as haplotypes, and in case of strong LD haplotype, frequencies have to be calculated directly from the population data (Szibor *et al*., 2003). The aim of this study was to perform population analysis and estimate haplotypes frequencies in the population of northern Croatia (Varadinska, Medimurska, Koprivnicko-Krievacka and Krapinsko-Zagorska counties). Although this region has been historically under strong influence of Hungary, we do not expect to find significant differences in haplotype frequencies from eight X-STRs typed in a previously published study on a population of central Croatia (Grskovic *et al*., 2013), due to migration processes between these two regions caused by Homeland War in 1990s.

## Materials and Methods

### Subjects

In total, 202 samples (from 102 males and 100 females) from northern Croatia were analyzed. Participants were unrelated and were selected from all four counties in an attempt to account for all subpopulation variations. All samples were collected during routine forensic work by the Forensic Science Centre "Ivan Vučetić", and their use in the study was approved by the Ethics Committee of the Institute for Medical Research and Occupational Health, Zagreb, Croatia.

### DNA extraction and Argus X-12 genotyping

Samples were extracted from FTA (Flinders Technology Associates) cards (Whatman, Maidstone, Kent, UK) using Chelex 100 (Walsh *et al*., 1991). For quantification, we used the Investigator Quantiplex Kit (Qiagen GmbH, Hilden, Germany) and all samples were normalized to approximately 1 ng/μL. Investigator® Argus X-12 Kit (Qiagen GmbH, Hilden, Germany) was used for amplification of 12 X-STR markers, and amelogenin for sex determination. Amplification products were analyzed on 3500 Genetic Analyzer (Applied Biosystems, Foster City, CA, USA). Data obtained from capillary electrophoresis were analyzed using GeneMapper® ID-X software (version 1.4, Applied Biosystems). All procedures and protocols were carried out following manufacturer's instructions.

### Allele frequency and population genetics analysis

The allele frequencies in both male and female samples, and haplotype frequencies in male samples were determined by counting. Female samples were tested for Hardy-Weinberg Equilibrium (HWE), including observed heterozygosity (Ho) and expected heterozygosity (He). The presence of pairwise linkage disequilibrium (LD) between loci was tested by the exact test using a Markov chain for male, and by likelihood ratio test using the Expectation-Maximization algorithm for female samples. Pair-wise genetic distances (F_ST_) were calculated for inter-population comparison of haplotype frequencies between northern Croatia and Italian (Bini *et al*., 2015), western Mediterranean (Ferragut *et al*., 2015), Czech (Zidkova *et al*., 2014), German (Edelmann *et al*., 2012), Hungarian (Horvath *et al*., 2012), Swedish (Tillmar, 2012), Danish, Somalian, Greenlandic (Tomas *et al*., 2012), Chinese and Japanese populations (Uchigasaki *et al*., 2013). Comparison with a population of Central Croatia was based on allele frequencies of DXS10135, DXS8378, DXS7132, DXS10074, HPRTB, DXS10101, DXS10134 and DXS7423 loci (Grskovic *et al*., 2013), while comparison with Bosnian-Herzegovinian population was based on DXS8378, DXS7132, HPRTB, and DXS7423 loci (Diegoli *et al*., 2011). Genetic heterogeneity within population was estimated as gene diversity for male haplotype data. All computations were performed using Arlequin software v3.5.2.2 (Excoffier and Lischer, 2010) and significance level for all statistical tests was set to 0.05, corrected for multiple comparisons using Bonferroni adjustment.

Forensic parameters such as polymorphism information content (PIC), power of exclusion (PE), power of discrimination (PD) for males and for females, mean exclusion chance (MEC) for deficiency cases (Krüger's formula), MEC for normal trios consisting of a mother, a daughter and a putative father (Kishida's formula), and MEC for duos consisting of a daughter and a putative father (Desmarais' formula), were computed using on-line application at ChrX-STR.org web page. Some parameters were also computed with male haplotype frequencies using supplementary R script (Zidkova *et al*., 2014).

## Results and Discussion

Allele frequencies, Ho and He, and P values for the HWE of 12 X-STR in the population of northern Croatia were determined (Table S1). Variant alleles were observed at the DXS10148 (14, 17, 25, 26, 28, 28.2, 31.1), DXS10134 (36.2, 36.3), DXS10146 (29.1, 37.2) and HPRTB (11.2) loci. A null allele was observed in one male sample on DXS10148 locus.

The most informative marker for the northern Croatia region was DXS10135 (PIC = 0.9211) (Table 1). This marker had a total of 27 alleles, of which allele 23 was the most frequent (0.1325). The least polymorphic marker was DXS8378 (PIC = 0.6275) with six alleles, and the added frequency of the 10, 11 and 12 allele was 0.937 (Table S1). No statistically significant deviations from HWE were observed at any locus (P=0.004 after Bonferroni correction). In female samples, significant LD (P < 0.001) was observed only within LG3 (DXS10101–DXS10103). The LD test in male samples showed no correlation between markers in any LG.

**Table 1:** Forensic parameters for 12 X-STR markers.

Linkage Group	Locus	No. of alleles	PIC	PE	PD female	PD male	MEC Krüger	MEC Kishida	MEC Desmarais Duo
LG1	DXS10148	20	0.872769	0.762610	0.975410	0.883874	0.766053	0.872549	0.783924
	DXS10135	27	0.921104	0.848577	0.989712	0.925902	0.850060	0.920891	0.858587
	DXS8378	6	0.627503	0.406584	0.842994	0.685765	0.422689	0.627382	0.481646
LG2	DXS7132	7	0.710683	0.512814	0.897290	0.751779	0.523731	0.710565	0.573043
	DXS10079	10	0.790556	0.626911	0.941417	0.814863	0.637918	0.790556	0.671062
	DXS10074	12	0.802868	0.645589	0.947461	0.824664	0.656998	0.802755	0.687303
LG3	DXS10103	7	0.693019	0.475032	0.890440	0.729302	0.511325	0.693019	0.553034
	HPRTB	9	0.709105	0.505022	0.897987	0.747220	0.528431	0.709105	0.572241
	DXS10101	19	0.887063	0.787395	0.980193	0.896069	0.790727	0.887063	0.805366
LG4	DXS10146	22	0.886202	0.785306	0.980142	0.895044	0.789914	0.885766	0.804508
	DXS10134	22	0.834844	0.697404	0.961441	0.851288	0.706512	0.834620	0.730658
	DXS7423	5	0.667246	0.453322	0.870623	0.715923	0.472121	0.667246	0.524531
			2.81712E-09	2.69609E-06	2.7537E-16	6.86998E-10	1.88019E-06	2.84802E-09	6.48025E-07
Combined			0.99999999718287	0.99999730390715	1.00000000000000	0.99999999931300	0.99999811981260	0.99999999715197	0.99999935197486

Abbreviations: PIC - polymorphism information content; PE - power of exclusion; PD - power of discrimination; MEC - mean exclusion chance.

Haplotype frequencies for each LG were calculated. There were 89, 72, 73, and 86 haplotypes detected in LG1, LG2, LG3, and LG4, respectively (Table S2). Proportions of the observed haplotypes among the number of possible haplotypes varied from 2.74 to 8.57% across all LGs. LG1 was the most informative, with a frequency of 0.0297 for the most common haplotype, whereas LG2 was the least informative, with a frequency of 0.049 for the most common haplotype. Gene diversity values of 0.9974 for LG1 and 0,9915 for LG2 support these findings. Forensic parameters were calculated for each marker separately. Combined values including all loci were also computed. Combined power of discrimination yielded high values, exceeding 0.999999999, in both male and female samples (Table 1).

We compared haplotype frequencies with eleven other populations, including Czech, German, Italian, western Mediterranean (pooled populations of Valencia, Majorca and Minorca), Hungarian, Swedish, Danish, Somali, Greenlandic, Chinese and Japanese populations. A pair-wise genetic distances (F_ST_) were calculated, with differences considered statistically significant for P-values < 0.001 after Bonferroni correction. As expected, there were no significant differences between the northern Croatia population and other European populations, which was also observed when comparing allele frequencies with the population of central Croatia and of Bosnia and Herzegovina. Significant differences in all four LGs were detected only for the Greenlandic population, which is expected, since it has been shown that the Greenland population is genetically different from other European and Asian populations (Tomas *et al*., 2012). The northern Croatia population differed from Somali, Chinese and Japanese populations only in certain LGs. Ethnic differences, as well as diverse sample sizes could explain this result. It has been shown that genetic variability between populations increases with the increase of the geographical distance (Tomas *et al*., 2012), which is in concordance with our results.

No significant differences were observed between our results and a previous study on 8 X-STRs in central Croatia population (Grskovic *et al*., 2013).

In conclusion, application of the Investigator® Argus X-12 Kit for analysis of the northern Croatia population showed that all X-STR markers are in HWE and can thus be used for match probability calculations. This kit should also be suitable for forensic casework because of the highly polymorphic markers and the high PD exhibited for the population of northern Croatia.

## Supplementary material

The following online material is available for this article:

Table S1 - Allele frequencies for 12 X-STRs in northern Croatia population

Table S2 - Haplotype frequencies for 12 X-STRs markers in four linkage groups
