# Supplementary material for: Investigator Argus X-12 study on the population of northern Croatia
Source: Genet Mol Biol. 2016 Oct 20;40(1):80–3. doi: 10.1590/1678-4685-GMB-2015-0261 (PMC5409765; doi:10.1590/1678-4685-GMB-2015-0261)
Supplement: Supplementary file 1 [file 1415-4757-gmb-1678-4685-GMB-2015-0261-Suppl01.pdf]

Table S1 - Allele frequencies for 12 X-STRs in northern Croatia population, N=202

		LG1			LG2			LG3			LG4		
**Allele**	**DXS10148**	**DXS10135**	**DXS8378**	**DXS7132**	**DXS10079**	**DXS10074**	**DXS10103**	**HPRTB**	**DXS10101**	**DXS10146**	**DXS10134**	**DXS7423**	**Allele**
**7**						0.0563							**7**
**8**						0.1391							**8**
**9**			0.0265			0.0099		0.0166					**9**
**10**			0.3079					0.0066					**10**
**11**			0.4139	0.0132		0.0033		0.1093					**11**
**11.2**								0.0033					**11.2**
**12**			0.2152	0.1358				0.3709					**12**
**13**			0.0331	0.2881		0.0099		0.2815				0.1258	**13**
**14**	0.0066			0.3311	0.0033	0.0066		0.1457				0.3245	**14**
**15**			0.0033	0.1887	0.0166	0.0828	0.0099	0.0464				0.3775	**15**
**16**		0.0066		0.0364	0.0397	0.2616	0.0960	0.0199		0.0033		0.1391	**16**
**17**	0.0199	0.0099		0.0066	0.0828	0.2450	0.0662					0.0331	**17**
**17.1**		0.0033											**17.1**
**18**	0.1196	0.0099			0.1623	0.1225	0.1854						**18**
**19**	0.0199	0.0397			0.2450	0.0430	0.4305						**19**
**19.1**		0.0166											**19.1**
**20**	0.0033	0.0397			0.2715	0.0199	0.1921						**20**
**20.1**		0.0232											**20.1**
**21**		0.1060			0.1159		0.0199						**21**
**21.1**		0.0033											**21.1**
**22**		0.0662			0.0530								**22**
**22.1**	0.0033	0.0066											**22.1**
**23**	0.0399	0.1325			0.0099								**23**
**23.1**	0.0465	0.0033											**23.1**
**24**	0.0266	0.0695								0.0132			**24**
**24.1**	0.1462												**24.1**

		LG1			LG2			LG3			LG4		
**Allele**	**DXS10148**	**DXS10135**	**DXS8378**	**DXS7132**	**DXS10079**	**DXS10074**	**DXS10103**	**HPRTB**	**DXS10101**	**DXS10146**	**DXS10134**	**DXS7423**	**Allele**
**25**	0.0033	0.1026								0.0695	0.0033		**25**
**25.1**	0.1728												**25.1**
**25.2**									0.0166				**25.2**
**26**	0.0066	0.0861								0.1656			**26**
**26.1**	0.1429	0.0033											**26.1**
**26.2**									0.0132				**26.2**
**27**		0.0464							0.0132	0.0993			**27**
**27.1**	0.1395												**27.1**
**27.2**									0.0530				**27.2**
**28**	0.0033	0.0530							0.0199	0.1589			**28**
**28.1**	0.0698												**28.1**
**28.2**	0.0033								0.1192				**28.2**
**29**		0.0662							0.0166	0.1589			**29**
**29.1**	0.0199	0.0033								0.0033			**29.1**
**29.2**									0.1325				**29.2**
**30**		0.0596							0.0464	0.0629			**30**
**30.2**									0.1457				**30.2**
**31**		0.0298							0.0497	0.0397	0.0199		**31**
**31.1**	0.0066												**31.1**
**31.2**									0.1556				**31.2**
**32**		0.0033							0.1225	0.0066	0.0066		**32**
**32.2**									0.0464				**32.2**
**33**		0.0066							0.0232		0.0662		**33**
**33.2**									0.0033	0.0033			**33.2**
**34**		0.0033							0.0166		0.1788		**34**
**34.2**									0.0033				**34.2**
**35**									0.0033		0.1391		**35**

		LG1			LG2			LG3			LG4		
**Allele**	**DXS10148**	**DXS10135**	**DXS8378**	**DXS7132**	**DXS10079**	**DXS10074**	**DXS10103**	**HPRTB**	**DXS10101**	**DXS10146**	**DXS10134**	**DXS7423**	**Allele**
**36**											0.2384		**36**
**36.2**											0.0033		**36.2**
**36.3**											0.0033		**36.3**
**37**											0.1788		**37**
**37.2**										0.0066	0.0099		**37.2**
**38**											0.0397		**38**
**38.3**											0.0066		**38.3**
**39**											0.0298		**39**
**39.2**										0.0397			**39.2**
**39.3**											0.0132		**39.3**
**40**											0.0033		**40**
**40.2**										0.0232			**40.2**
**40.3**											0.0166		**40.3**
**41.1**											0.0033		**41.1**
**41.2**										0.0099			**41.2**
**41.3**											0.0232		**41.3**
**42.2**										0.0166			**42.2**
**42.3**											0.0099		**42.3**
**43.2**										0.0364			**43.2**
**43.3**											0.0033		**43.3**
**44.2**										0.0364	0.0033		**44.2**
**44.3**													**44.3**
**45.2**										0.0066			**45.2**
**46**										0.0033			**46**
**46.2**										0.0364			**46.2**
Ho	0.8600	0.9500	0.6800	0.7600	0.8300	0.7900	0.7200	0.7900	0.8800	0.8800	0.8500	0.7000	Ho
He	0.8913	0.9269	0.6884	0.7484	0.8257	0.8174	0.7355	0.7567	0.8955	0.8927	0.8358	0.7186	He

	LG1				LG2			LG3			LG4		
**Allele**	**DXS10148**	**DXS10135**	**DXS8378**	**DXS7132**	**DXS10079**	**DXS10074**	**DXS10103**	**HPRTB**	**DXS10101**	**DXS10146**	**DXS10134**	**DXS7423**	**Allele**
P	0.3332	0.1579	0.8521	0.8068	0.4302	0.8783	0.2821	0.4568	0.4931	0.6272	0.2585	0.2865	*P*
SD	0.0003	0.0001	0.0003	0.0004	0.0004	0.0003	0.0005	0.0004	0.0004	0.0003	0.0002	0.0004	SD

Abbreviations: Ho - observed heterozygosity; He - expected heterozygosity; *P*-value - result of Hardy-Weinberg equilibrium test with a significance level 0.004; SD - standard deviation

No statistically significant deviation from HWE was observed in any locus.
