# Supplementary material for: Investigator Argus X-12 study on the population of northern Croatia
Source: Genet Mol Biol. 2016 Oct 20;40(1):80–3. doi: 10.1590/1678-4685-GMB-2015-0261 (PMC5409765; doi:10.1590/1678-4685-GMB-2015-0261)
Supplement: Supplementary file 2 [file 1415-4757-gmb-1678-4685-GMB-2015-0261-Suppl02.pdf]

Table S2 - Haplotype frequencies for 12 X-STRs markers in four linkage groups, N=102.

LG1					LG2					LG3					LG4				
DXS10148	DXS10135	DXS8378	n	frequency	DXS7132	DXS10079	DXS10074	n	frequency	DXS10103	HPRTB	DXS10101	n	frequency	DXS10146	DXS10134	DXS7423	n	frequency
17	29	11	1	0.0099	11	19	16	1	0.0098	15	14	34	1	0.0098	25	33	14	1	0.0098
17	30	9	1	0.0099	12	17	19	1	0.0098	16	12	27.2	1	0.0098	25	36	14	1	0.0098
17	31	11	1	0.0099	12	18	18	1	0.0098	16	12	28.2	1	0.0098	25	36	15	1	0.0098
18	21	10	1	0.0099	12	18	19	1	0.0098	16	12	30	2	0.0196	25	36.2	14	1	0.0098
18	21	11	1	0.0099	12	19	16	1	0.0098	16	12	31	1	0.0098	25	37	14	2	0.0196
18	24	12	1	0.0099	12	20	7	1	0.0098	16	13	25.2	1	0.0098	25	38	13	1	0.0098
18	26	11	2	0.0198	12	20	15	1	0.0098	16	13	31.2	1	0.0098	25	38	15	1	0.0098
18	27	12	1	0.0099	12	20	16	3	0.0294	16	13	32	2	0.0196	26	31	15	1	0.0098
18	28	10	1	0.0099	12	20	19	1	0.0098	16	14	32	1	0.0098	26	34	14	2	0.0196
18	28	11	1	0.0099	12	21	18	1	0.0098	17	11	29.2	1	0.0098	26	35	13	1	0.0098
18	30	12	2	0.0198	12	22	19	1	0.0098	17	12	29.2	1	0.0098	26	35	15	2	0.0196
19	23	11	1	0.0099	12	23	16	1	0.0098	17	12	31	1	0.0098	26	36	13	1	0.0098
19	33	12	1	0.0099	13	16	16	1	0.0098	17	12	32	1	0.0098	26	36	14	1	0.0098
23	21	11	1	0.0099	13	16	17	1	0.0098	17	13	31	1	0.0098	26	36	15	3	0.0294
23.1	21	10	1	0.0099	13	17	15	1	0.0098	17	13	34	1	0.0098	26	36	16	1	0.0098
23.1	24	11	1	0.0099	13	18	7	1	0.0098	17	14	32	1	0.0098	26	37	14	1	0.0098
23.1	24	12	1	0.0099	13	19	8	1	0.0098	17	15	32	1	0.0098	26	38	14	1	0.0098
23.1	26	10	1	0.0099	13	19	9	1	0.0098	18	9	30.2	3	0.0294	27	31	15	1	0.0098
23.1	29	11	2	0.0198	13	19	11	1	0.0098	18	11	28.2	1	0.0098	27	34	16	2	0.0196
24	22	10	1	0.0099	13	19	16	5	0.0490	18	11	29.2	1	0.0098	27	35	13	1	0.0098
24.1	19	10	1	0.0099	13	19	18	2	0.0196	18	12	27.2	1	0.0098	27	35	15	1	0.0098
24.1	21	9	1	0.0099	13	20	7	4	0.0392	18	12	28	1	0.0098	27	35	16	1	0.0098
24.1	21	11	2	0.0198	13	20	8	1	0.0098	18	12	29	1	0.0098	27	36	14	1	0.0098
24.1	21	13	1	0.0099	13	20	16	2	0.0196	18	12	30.2	1	0.0098	27	37	15	1	0.0098
24.1	22	9	1	0.0099	13	20	17	2	0.0196	18	12	31	1	0.0098	27	37	16	1	0.0098
24.1	22	10	1	0.0099	13	21	16	2	0.0196	18	12	31.2	1	0.0098	27	37.2	15	1	0.0098

LG1					LG2					LG3					LG4				
**DXS10148**	**DXS10135**	**DXS8378**	**n**	**frequency**	**DXS7132**	**DXS10079**	**DXS10074**	**n**	**frequency**	**DXS10103**	**HPRTB**	**DXS10101**	**n**	**frequency**	**DXS10146**	**DXS10134**	**DXS7423**	**n**	**frequency**
24.1	23	10	1	0.0099	13	22	17	1	0.0098	18	12	32.2	1	0.0098	28	31	14	1	0.0098
24.1	23	11	1	0.0099	13	23	17	1	0.0098	18	13	27.2	1	0.0098	28	31	15	1	0.0098
24.1	24	10	1	0.0099	14	16	17	1	0.0098	18	13	28.2	1	0.0098	28	32	16	1	0.0098
24.1	24	11	1	0.0099	14	17	8	2	0.0196	18	13	30	1	0.0098	28	33	13	1	0.0098
24.1	24	12	1	0.0099	14	17	18	1	0.0098	18	13	32	1	0.0098	28	34	14	2	0.0196
24.1	25	11	1	0.0099	14	18	8	1	0.0098	18	13	34	2	0.0196	28	36	14	1	0.0098
24.1	27	11	1	0.0099	14	18	17	1	0.0098	18	13	35	1	0.0098	28	36	15	2	0.0196
24.1	31	11	2	0.0198	14	18	18	1	0.0098	18	15	31.2	1	0.0098	28	37	15	1	0.0098
24.1	34	10	1	0.0099	14	19	8	1	0.0098	19	11	27.2	2	0.0196	28	37.2	14	1	0.0098
25.1	16	12	1	0.0099	14	19	9	1	0.0098	19	11	28.2	4	0.0392	28	38	15	1	0.0098
25.1	19	11	1	0.0099	14	19	16	2	0.0196	19	11	29.2	2	0.0196	28	39	15	1	0.0098
25.1	19.1	10	2	0.0198	14	19	17	4	0.0392	19	11	30.2	2	0.0196	28	41.3	17	1	0.0098
25.1	20	10	1	0.0099	14	19	18	1	0.0098	19	12	26.2	1	0.0098	29	32	16	1	0.0098
25.1	20.1	11	1	0.0099	14	19	19	1	0.0098	19	12	27	2	0.0196	29	33	13	1	0.0098
25.1	21	12	1	0.0099	14	19	20	1	0.0098	19	12	27.2	1	0.0098	29	34	13	1	0.0098
25.1	22	9	2	0.0198	14	20	7	1	0.0098	19	12	28.2	3	0.0294	29	34	14	3	0.0294
25.1	23	11	1	0.0099	14	20	16	2	0.0196	19	12	29.2	3	0.0294	29	34	15	2	0.0196
25.1	25	11	1	0.0099	14	20	17	1	0.0098	19	12	30	2	0.0196	29	34	16	1	0.0098
25.1	25	12	1	0.0099	14	20	18	1	0.0098	19	12	30.2	4	0.0392	29	35	14	1	0.0098
25.1	27	10	1	0.0099	14	20	20	1	0.0098	19	12	31.2	3	0.0294	29	35	15	2	0.0196
25.1	27	12	1	0.0099	14	21	8	1	0.0098	19	12	32.2	2	0.0196	29	36	16	1	0.0098
25.1	28	11	1	0.0099	14	21	15	2	0.0196	19	13	29.2	2	0.0196	29	36	17	1	0.0098
25.1	29	10	1	0.0099	14	21	17	1	0.0098	19	13	30.2	3	0.0294	29	37	16	1	0.0098
25.1	29	11	1	0.0099	14	21	18	2	0.0196	19	13	31	1	0.0098	29	39	17	2	0.0196
25.1	30	11	1	0.0099	14	22	17	1	0.0098	19	13	31.2	1	0.0098	29	44.3	15	1	0.0098
25.1	31	12	1	0.0099	14	22	18	2	0.0196	19	13	32	1	0.0098	29.1	33	14	1	0.0098
26.1	17	12	1	0.0099	15	17	16	2	0.0196	19	13	32.2	2	0.0196	30	35	13	1	0.0098

LG1					LG2					LG3					LG4				
DXS10148	DXS10135	DXS8378	n	frequency	DXS7132	DXS10079	DXS10074	n	frequency	DXS10103	HPRTB	DXS10101	n	frequency	DXS10146	DXS10134	DXS7423	n	frequency
26.1	20	11	1	0.0099	15	18	7	1	0.0098	19	14	30	1	0.0098	30	36	14	2	0.0196
26.1	20.1	10	1	0.0099	15	18	8	2	0.0196	19	14	30.2	1	0.0098	30	37	15	1	0.0098
26.1	21	10	1	0.0099	15	18	16	1	0.0098	19	14	31.2	1	0.0098	31	35	14	1	0.0098
26.1	22	9	1	0.0099	15	18	17	1	0.0098	19	15	31	1	0.0098	31	36	14	1	0.0098
26.1	22	11	1	0.0099	15	19	7	1	0.0098	19	15	31.2	1	0.0098	31	36	15	1	0.0098
26.1	23	12	1	0.0099	15	19	8	2	0.0196	20	11	29.2	1	0.0098	33.2	36	15	1	0.0098
26.1	25	10	2	0.0198	15	19	16	2	0.0196	20	11	30.2	1	0.0098	37.2	39.3	16	1	0.0098
26.1	25	11	1	0.0099	15	19	18	2	0.0196	20	12	27.2	1	0.0098	39.2	34	14	1	0.0098
26.1	25	13	1	0.0099	15	20	16	2	0.0196	20	12	28.2	1	0.0098	39.2	34	16	1	0.0098
26.1	26	13	1	0.0099	15	20	17	3	0.0294	20	12	30.2	1	0.0098	39.2	35	16	1	0.0098
26.1	27	11	1	0.0099	15	20	20	1	0.0098	20	12	31.2	3	0.0294	39.2	36	15	1	0.0098
26.1	29	11	1	0.0099	15	22	15	1	0.0098	20	13	28.2	1	0.0098	39.2	39.3	15	3	0.0294
26.1	30	10	1	0.0099	16	18	9	1	0.0098	20	13	30.2	1	0.0098	39.2	41.3	15	1	0.0098
26.1	30	11	1	0.0099	16	19	16	1	0.0098	20	13	32.2	1	0.0098	39.2	41.3	16	1	0.0098
27.1	18	11	1	0.0099	16	19	17	1	0.0098	20	14	28	1	0.0098	40.2	34	15	1	0.0098
27.1	21	10	1	0.0099	16	20	17	1	0.0098	20	14	31	1	0.0098	40.2	36	14	1	0.0098
27.1	21	12	1	0.0099	16	21	19	1	0.0098	20	15	29.2	1	0.0098	40.2	39	13	1	0.0098
27.1	23	11	3	0.0297	17	17	8	1	0.0098	20	16	32	1	0.0098	40.2	41.3	15	1	0.0098
27.1	25	11	2	0.0198	17	17	16	1	0.0098	21	13	29.2	1	0.0098	41.2	36	16	1	0.0098
27.1	26	11	1	0.0099						21	13	31.2	1	0.0098	41.2	40.3	14	1	0.0098
27.1	28	10	1	0.0099											42.2	25	15	1	0.0098
27.1	30	11	1	0.0099											42.2	34	13	1	0.0098
27.1	33	12	1	0.0099											43.2	33	15	1	0.0098
28.1	19.1	12	1	0.0099											43.2	36	14	1	0.0098
28.1	21	10	1	0.0099											43.2	42.3	15	1	0.0098
28.1	21	11	1	0.0099											44.2	33	16	1	0.0098
28.1	22	11	1	0.0099											44.2	34	16	1	0.0098

LG1					LG2					LG3					LG4				
**DXS10148**	**DXS10135**	**DXS8378**	**n**	**frequency**	**DXS7132**	**DXS10079**	**DXS10074**	**n**	**frequency**	**DXS10103**	**HPRTB**	**DXS10101**	**n**	**frequency**	**DXS10146**	**DXS10134**	**DXS7423**	**n**	**frequency**
28.1	23	10	1	0.0099											44.2	35	15	1	0.0098
28.1	23	11	1	0.0099											45.2	33	15	1	0.0098
28.1	24	10	1	0.0099											45.2	35	15	1	0.0098
28.1	24	11	1	0.0099											46.2	35	14	1	0.0098
28.1	27	10	2	0.0198											46.2	37	15	1	0.0098
29.1	22	10	1	0.0099											46.2	38	15	1	0.0098
29.1	25	10	1	0.0099															
29.1	29	11	1	0.0099															
31.1	21	10	1	0.0099															
Observed haplotypes			89					72					73					86	
Possible haplotypes			3240					840					1197					2420	
Gene diversity			0.9974					0.9915					0.9920					0.9963	
SD			0.0018					0.0029					0.0027					0.0020	
